# Venture capital, control rights, and family enterprise growth

**DOI:** 10.1371/journal.pone.0256318

**Published:** 2021-08-18

**Authors:** Xianjun Pang, Liping Liu

**Affiliations:** 1 Ankang University-Shaannan Eco-economy Research Centre, Ankang, China; 2 School of Business, Nankai University, Tianjin, China; 3 School of Economics and Management, Guangxi Normal University, Guilin, China; 4 International College, National Institute of Development Administration, Bangkok, Thailand; University of Salento, ITALY

## Abstract

This research explores and explains the path of family enterprise venture capital equity financing from the perspective of endogenous family control rights. We adopted unbalanced panel data on Chinese listed companies from 2007 to 2018. Empirical research shows that there are significant differences in the impact of venture capital on the growth performance of family enterprises and non-family enterprises. Venture capital negatively affects the growth performance of family enterprises, while the negative impact of venture capital on family enterprises is not significant. In addition, family control positively moderates the negative impact of venture capital on family enterprise growth performance.

## Introduction

In the context of China’s economic transformation, family-controlled enterprises play an important role. With the increase in the development of family enterprises, more and more of them are seeking external financing. According to statistics from PwC’s 2018 *Global Family Enterprises Research Report*, 93% of family enterprises will achieve transformation and growth through external financing in the future. Among them, venture capital is a good equity financing method for family enterprises [1]. Venture capital can not only ease the financing constraints of enterprises but also provide other non-capital appreciation services such as management support so as to help enterprises grow [2–4]. Family enterprises will embrace venture capital due to financing constraints and growth transformation [5], and venture capital investment in family enterprises has become a remarkable phenomenon [6].

However, at present, there are few research results on the external equity financing of venture capital with family enterprises, a topic that needs to be tackled through theoretical research. Moreover, as an organizational form with dual characteristics of enterprise and family, family enterprises have unique control characteristics, which are highlighted by the degree of family control in different family enterprises. Therefore, family enterprises are affected by the degree of family control in the process of venture capital financing, which makes the problem of venture capital financing for family enterprises different from ordinary enterprises. In addition, venture capital firms are more focused on short-term benefits and financial returns, while family enterprises are more focused on longevity and social emotional wealth [7]. Thus, in China’s immature capital market, does venture capital have an impact on the growth and transformation of family enterprises? And is this impact different under family control? What causes any differences? How people should coordinate the interest relationship between family enterprises and venture capital firms to achieve the goal of win-win cooperation is a question worth exploring. In particular, the influence of the degree of family control on the growth performance of venture capital involvement in family enterprises also highlights the characteristics of family enterprises that distinguish them from non-family enterprises. Examining the effect of venture capital financing in family enterprises from the perspective of family enterprise heterogeneity is helpful to explore and explain the influence of venture capital on the growth of family enterprises.

This article’s possible contributions are as follows: first, studies of venture capital in family enterprise research are still in their infancy [8], and the present theory based on the assumption of homogeneity [9] does not sufficiently cover family enterprise features in research. As the resources and organizational structure of family enterprises are special, it is particularly important to compare the behaviours of family enterprises and non-family enterprises. Second, organizational structure is very important for enterprises. Organizational structure represents a type of internal environment that can promote and hinder the management process. Therefore, the influence of external equity financing from venture capital on enterprise growth will be constrained by the organizational control characteristics of the enterprise itself. Third, unlike previous literature, this paper considers family control and its control degree, and through comparison and analysis with non-family enterprise venture capital effects on family enterprises and testing of how family control in venture capital affects the role of family enterprises, explores and explains the role of family enterprise venture capital with an emphasis on equity financing. In other words, this study provides a clearer understanding of the economic consequences of venture capital equity financing of family enterprises, and the research findings also highlight practical considerations that family enterprises can introduce to their venture capital decision-making.

This paper is constructed as follows. First, we review the literature on venture capital, control rights, and family enterprise growth, and put forward the research hypotheses. Second, we explain the research method and data collection processes. Third, we present the research results. Finally, we provide theoretical and practical implications.

## Theoretical analysis and hypothesis presentation

### Venture capital shareholding and family enterprise performance

At present, more and more family enterprises are beginning to introduce strategic investors such as venture capital firms to help them complete strategic transformation and inheritance. However, when venture capital is involved in these family enterprises, does it hinder or promote their growth and performance?

First, the intervention of venture capital in family enterprises changes the centralized authoritative governance structure of traditional family enterprises, leading to conflict between family enterprises and venture capital. For example, in 2009, Mr. Xiao Chen signed a financing agreement with Bain Capital that sparked a battle for control the following year. In 2008, the company South Beauty successfully introduced financing from CDH Investments and the International Capital Corporation by selling 10% of its shares. However, in 2011, there was a fierce conflict between CDH Investments and the International Capital Corporation that led to a decline in corporate performance. In 2012, the company Lei-Shi Lighting and major shareholders from different investment institutions staged a forceful drama over the business. The heterogeneity of these family enterprises often leads to the exclusion of venture capital firms from decision-making and the introduction of contradictions, indicating that there is a contradictory and conflicting relationship between family enterprises and venture capital firms. Second, venture capital promotes enterprise performance through meaningful cooperation with family enterprises. For example, in 2007, the company Daphne saw a decline in performance. After introducing venture capital, Daphne survived the crisis with a 29% increase in turnover and a 56.54% increase in net profit. In 2009, the founder of Natural Beauty, Yanping Cai, brought the Carlyle Group in for the long-term development of the company, and Natural Beauty’s sales revenue increased by 8.4%.

From the perspective of theoretical research, most existing studies have focused on the influence of venture capital financing on family enterprise governance and the concept of “defamily”, but rarely have these studies extended to the impact of these influences on the growth and performance of family enterprises. A small number of relevant studies, such as those on the role of venture capital in family enterprise succession, have great implications for this study. The inheritance of ownership and management rights are the biggest challenges for family enterprises. Succession in many family enterprises is not handled well [10, 11]. Venture capital can be conducive to the generation of inheritance for family enterprises, and management buyouts and buy-ins are becoming the solutions of choice for ownership inheritance in family enterprises [12]. In addition, the overly complex ownership structure of family enterprises will affect their long-term efficiency, and to reduce the complexity of ownership, the original shareholders need to withdraw part or all of their capital, which can be achieved through the purchase of controlling shares by venture capital firms. Therefore, venture capital is involved in helping family enterprises transform their organizational governance and ownership so that they can continue to thrive. When a family member or part of a family sells their stake to a venture capital firm, there is a change in control of the family because the pressure from venture capital investment managers will also lead to changes in the structure of the organization and its management.

However, the impact of any venture capital may be influenced by the specific characteristics of different family enterprises. When we analyse the influence of venture capital on family and non-family enterprises, it is often necessary to consider whether venture capital amplifies the family enterprises’ advantages and disadvantages. After receiving funds, the performance of the family enterprise depends largely on the supervision and guidance activities of the venture capital firm. A family enterprise’s proximity to venture capital, if only for financing purposes, may endanger the close relationship between the two since both the family and the non-family shareholders must participate to improve growth performance.

When a family enterprise seeks venture capital for financing, the reason for providing the capital itself is more closely related to the type of investor than the motivation of adding value. At this stage, family enterprises tend to be overconfident in their management culture while lacking the capital they need to invest in growth. As a result, family enterprises are reluctant to give up majority stakes in fundraising to maintain full control over their growth. But family enterprises may also get close to venture capital firms and allow some family shareholders to exit, often by ceding their minority stakes to the outside investors. In this way, the original family shareholders tend to uphold the company’s traditional management culture.

Whether they own a majority or minority stake in a family enterprise, venture capital firms carry out many other investment firm duties, including monitoring and value-adding activities, to help companies improve their performance. After the intervention of venture capital, two situations may occur: On the one hand, the conflict between the two management cultures will lead to the decrease of information flow between a family enterprise and a venture capital firm, thus restricting the venture capital firm’s ability to identify and provide solutions to daily operation problems. On the other hand, the venture capital firm may accept the dominance of existing management cultures and give up its ability to add value. Therefore, the venture capital firm’s ability to implement its management culture in the invested enterprise, or its conflict with the existing management culture, may affect the growth performance of the enterprise since power dynamics in family enterprises are different from that of non-family enterprises.

There are differences between the goals of venture capital firms and those of family enterprises, and the management culture of family enterprises is notoriously deep-rooted and difficult to change [13].

Therefore, this paper hypothesizes the following.

Hypothesis 1: The influence of venture capital on the growth performance of family and non-family enterprises is different.

On the one hand, the positive role of venture capital financing of family enterprises is mainly reflected in the following three ways.

First, venture capital can encourage family enterprises to take risks and improve their enterprise growth performance. To develop a family enterprise, it is necessary to choose a high-risk and high-yield investment project. Risk-taking reflects the willingness of enterprises to pursue high profits and pay for them [14]. Among the factors that promote the increase of enterprise performance, risk-taking is an important consideration [15]. However, the nature of enterprise ownership can affect enterprise risk-taking [16], and family enterprises tend to avoid risks [17, 18]. Family enterprises are more risk-averse than non-family enterprises because family owners keep most of their wealth in one asset. Wright, Ferris [19] found that the shareholding ratio of institutional investors was positively correlated with enterprise risk-taking and that the introduction of risk capital would improve the risk-taking level of family enterprises. Risky projects promote the capital accumulation, technological progress, and core competitiveness of family enterprises, thus producing good performance. Therefore, venture capital can promote an enterprise’s risk-taking, making the enterprise change its inherent business model and realize rapid growth.

Second, venture capital can enhance the level of professionalism of enterprise management and improve the science of decision-making. According to Fan and Gu [20], on nearly 500 private enterprises, the employment of family members in enterprises that introduce venture capital is significantly reduced. Venture capital can not only relieve the capital needs of enterprises but also help entrepreneurial family members to exit their stocks, reduce the tenure of family members, and clear obstacles with personnel to encourage the smooth succession of the second generation of the rich. After the introduction of venture capital, when the proportion of venture capital equity is high enough to influence the enterprise’s management decisions, it is possible for a venture capital firm to transform a family enterprise according to the firm’s own management culture. Zata Poutziouris [21] found that growth-oriented family enterprises are more likely to benefit from the absorption of venture capital, which improves the growth performance of enterprises by optimizing management and decision-making.

Third, venture capital can ease the resource constraints of family enterprises and help them grow rapidly. At present, the lack of capital, talent, technology, and other resources has become a major obstacle to the growth of family enterprises. Venture capital can solve the financial problem associated with the growth of a family enterprise at the same time that other improvements are made. A venture capital firm can also use its own social network to bring advanced technology, excellent management personnel, and mature business concepts so as to overcome the resource constraints of a family enterprise and improve its growth performance.

In addition, according to the resource-based theory, a company’s ability derives from its endowment of strategic resources [22]. The successful implementation of an initial public offering (IPO) is considered to be the embodiment of a company’s ability [23]. Family enterprises need sufficient resource endowment to cultivate such an ability, and the IPO process is essentially a process of resource supply. Family enterprises generally lack the experience and ability to invest professionally in an IPO, so founder families tend to seek the resources from an outside party. Among them, the introduction of venture capital is the main form of resource withdrawal, and the investment of venture capital ultimately leads to better growth performance for family enterprises.

Venture capital intervention can help family enterprises to raise funds, reduce the complexity of ownership, improve the level of risk-taking, successfully go public, and make appropriate changes in organization, management, corporate governance, and other aspects so as to improve their performance. Based on that, hypothesis 2a is proposed.

Hypothesis 2a: Venture capital shareholding can promote the growth and performance of family enterprises.

On the other hand, the negative effects of venture capital financing of family enterprises are mainly reflected in the following ways.

First, family control and governance also have competitive advantages. The introduction of venture capital may destroy the original competitive advantages that the enterprises had to some extent. For example, family enterprises may face an increase in agency costs brought by changes in the governance structure. According to Jensen Michael and Meckling William [24], agency costs represent the loss of enterprise value caused by agency conflicts. In addition, venture capital firm participation in the corporate governance of family enterprises is lower than in non-family enterprises, so the inhibitory effect on agency costs is less obvious in family enterprises than in non-family enterprises, which also results in the loss of enterprise growth performance. In other words, the increase in income from introducing venture capital may not be as high as the cost, which will restrain the family enterprise’s growth performance to some extent.

Second, when a venture capital firm is involved in a family enterprise, the heterogeneity between the controlling founding shareholders of the family enterprises and the venture capital shareholders in terms of risk appetite, shareholding purpose, holding time, and expected returns leads to conflicts between the founders and venture capital shareholders. The personal wealth of the founders of family enterprises is closely related to the business, which means that the founders need to exert strong control and influence on the business to protect the family’s and their own wealth. In terms of the decision-making body, the founder of a family enterprise is usually also the CEO, and CEOs will cultivate a team that obey them. Founders design organizational strategies and structures based on their rich experiences in the industry, which will promote the successful performance of family enterprises in the initial stage of business. Moreover, venture capital shareholders have dual identities; that is, they are the agents of their own investors and the clients of private listed companies. In the context of multiple principal-agent relationships, venture capital faces the dual pressure to obtain both short-term benefits and long-term reputation [25, 26].

Third, the growth and evolution of family enterprises are relatively gradual, while venture capital investment tends to cultivate the invested enterprises so that they grow rapidly in the shortest possible time. However, these two goals stand in contradiction. Moreover, in some cases, the introduction of venture capital can overcome the rigid management culture of family enterprises and make them last longer. Founders of family enterprises who want to keep their businesses going for generations will become more conservative in their decisions because the higher the level of risk a start-up takes, the greater the risk that its wealth will be destroyed and subsequently impact the established organizational culture. If external investors impose their own cultural concepts on family enterprises, they will run into conflicts with the family enterprises’ management cultures, which may have negative impacts on the performance of the family enterprises.

In short, venture capital may destroy the existing advantages of a family organization, resulting in increased agency costs, conflicts between venture capital investors and business owners, and inconsistent goals between them, and thus causing the negative effects of venture capital financing on family enterprises. Based on this, hypothesis 2b (the competing hypothesis of hypothesis 2a) is proposed.

Hypothesis 2b: Venture capital shareholding hinders the growth and performance of family enterprises.

### Degree of family control as a moderating effect

The difference in the degree of family control will have different levels of influence on the behaviour and venture capital of family enterprises. On the issue of venture capital financing of family enterprises, the specific institutional arrangement of the endogenous control of a family enterprise will have a different influence on the introduction of venture capital than with non-family enterprises.

First, the degree of family control will affect the main body of family enterprises. Many scholars have found that the degree of control has an effect on enterprise performance [27, 28]. Compared with non-family enterprises, family enterprises have more characteristics in the degree of complete separation. The characteristics of family enterprises are largely reflected in the level of control and the difference in the separation of control and cash flow rights [29]. Compared with non-family enterprises, listed family enterprises have an obvious two-way separation. The degree of family control is different, and the degree of separation of the two rights will be different after venture capital intervention. The degree of control will affect the degree of separation of the two rights after venture capital financing of family enterprises [30]. In addition, if the concentration of control in family enterprises is high, or even if there is the phenomenon of a single dominant share, then family shareholders with relative or absolute control will find it easier to hollow out the enterprise through a pyramidal shareholding structure and infringe on the interests of small and medium-size investors [31].

Second, the degree of family control will have different effects on the venture capital firm’s behaviour. A venture capital firm, as a special equity investor, has a special control right requirement over an enterprise. Venture capital data that has been used to test the financing contract theory, through the practice of comparing financing contracts with the characteristics of the financing theory of such contracts, has shown that the apparent characteristics of venture capital financing are that it allows the venture capitalist to independently configure cash flow rights, voting rights, directors, liquidation rights, anti-dilution rights, and other controlling factors [32]. Rights such as cash flow rights, voting rights, control rights, and future financing rights often rely on the observation of financial and non-financial performance. In order to reduce the problem of holding entrepreneurs and investors together, venture capital firms usually require non-compete clauses and equity transfer clauses. The demands of a venture capital firm subject to the control right and the game process with a family enterprise will be affected by the degree of the enterprise’s control right. Thus, the degree of control that reflects the enterprise’s characteristics will have an impact on the independent allocation of the control right of the venture capital firm, contractual terms, and other aspects of venture capital behaviour.

The difference in the degree of family control will have different influences on the main body behaviour and the venture capital of family enterprises and will ultimately affect how venture capital impacts family enterprises. Based on the above analysis, this study sought to explain the role of venture capital in family enterprises from the path of family control, took the degree of family control as a moderating variable, and proposed hypothesis 3.

Hypothesis 3: The degree of family control plays a moderating role in the influence of venture capital on the growth and performance of family enterprises.

Furthermore, family control is the basis for family enterprises to exercise their decision-making power [33]. The more centralized the family control is, the more singular their decision-making power and the higher the degree of power in the family enterprise. Moreover, the higher the degree of family control, the easier it will be for the controlling family to participate in business management and realize family supervision over the business [7]. When the enterprise’s performance is poor, especially in the face of operational difficulties, in order to avoid the threat of loss of control, the family controller will strengthen management and control over the enterprise’s business activities and try their best to give full attention to the subjective initiative. In such a situation, the difference between the relationships of family enterprises and venture capital firms will be more prominent. Therefore, we believe that compared with family enterprises with a weak degree of family control, venture capital intervention in family enterprises with a strong degree of family control will see a higher impact on their performance. Therefore, the following research hypothesis is proposed.

Hypothesis 4: The influence of venture capital on the growth and performance of family enterprises is positively regulated by the degree of family control. In other words, compared with family enterprises with strong degrees of control, family enterprises with weak degrees of control see more obvious positive effects from venture capital financing.

## Research methods

### Procedure

In this study, we selected the non-balanced panel data of the listed enterprises on the growth enterprise market with small and medium-size boards from 2007 to 2018, and the following samples were deleted: (1) Special treatment (ST) companies, (2) financial and insurance companies, and (3) companies with missing variables. After further combing the sample enterprises, 499 enterprises with venture capital investment and 639 enterprises without venture capital investment were obtained. Among them, there were 222 family enterprises (Fami = 1) and 916 non-family enterprises. The final data collected were 3,152 company-annual non-family sample observations and 843 company-annual family sample observations. In order to prevent the influence of outliers on the research conclusions due to the large sample size, the continuous variables in the sample were processed to 1% Winsorization.

After identification of the family enterprises through the Taian database according to each company’s prospectus, information about the main shareholder is introduced as well as data on the board of directors structure, the inspection control structure, and the ownership structure. Information was also gathered from the annual reports *Shareholder Relations or Concerted Action Person* and *Between the Company and the Actual Control of Property Rights and the Control Block Diagram* to determine the family members of the business enterprise. In addition, in order to understand the latest data on family enterprise owners and managers, securities software can be used to check a company’s profile and high-level governance and other information. Regarding suspected family members such as senior executives who have the same surname as the actual controller or relatives who have been identified as coming from the same place and so on, a multiparty search engine was used to determine whether there was any family relationship between senior executives and the actual controller. Among them, the relationship between family members may be father and son, mother and son, husband and wife, brothers, and sisters. Specific identification for the family enterprises grasped the characteristics of enterprise equity and management rights according to the following two conditions: First, the largest shareholder of the company is the entrepreneur or their family members and holds at least 10% of the shares. Second, the founders or family members of the company serve as directors and managers of the company. The identification of whether there is any risk capital, the shareholding ratio of risk capital, and the participation of venture capital in corporate governance mainly came from the CV Source database and refers to the prospectus and China venture capital development report for verification. The related data of the measurement indicators of family control degree (ultimate control and cash flow and the degree of excess control overboard seats) were manually calculated. The definition of the control right and cash flow right of a family enterprise matches that of Claessens, Djankov [34]. Most of the financial performance indicators came from the China Stock Market and Accounting Research Database and Wind databases, while corporate governance data came from the China Center for Economic Research database. Missing values were collected manually using various search engines to search resources such as annual reports and company websites.

### Measure

#### Variables

*Dependent variable*. Considering the particularity of family enterprises and the long-term effect of venture capital on such enterprises, the growth of sales revenue, the growth in the number of employees, and the growth of net profit were selected as the proxy indicators of growth performance.

*Independent variables*. The independent variable is venture capital. If the observation enterprise had no venture capital support, the value of venture capital support is 1; otherwise, it is zero.

*Moderating variables*. The moderating variables in this paper are two measures of the degree of family control: the degree of separation and control of cash flow rights (Seperati and Borderpro). We selected these two indicators as the proxy indicators to measure the degree of family control for the following reasons: Referring to the essence of family control expounded by other studies [35, 36]. Villalonga and Amit [36] measured the degree of family control using the abovementioned variables. In addition, the degree of control in practice is manifested in the general meeting of shareholders and the board of directors. Therefore, the separation degree of ultimate control right and cash flow right is taken as the measurement index at the shareholders’ meeting level. The family’s excess seats on the board of directors are used as the measurement index at the board level.

*Control variables*. In terms of control variables, factors such as the overall characteristics of the enterprise, the financial characteristics of the enterprise, and the characteristics of corporate governance were considered. The overall characteristics of the controlled enterprise include company size and enterprise age; the financial characteristics of the controlled enterprise include return on net assets and the asset-liability ratio [37]. The corporate governance characteristics of control include management shareholding, measured by the total number of shares held by all senior executives of the company [38], and board size.

#### Model design

(1) The basic regression model and (2) the moderating effect of the degree of family control were constructed to test the effect of venture capital on the growth performance of a family enterprise, and the influence of the degree of family control on this effect was further analysed. In the following model, the dependent variable (Growth) is informed by the three measurement indexes of the change in growth performance. The independent variable is the dummy variable (VC) showing whether a family enterprise is supported by venture capital. The interaction term is the cross of the venture capital and the degree of family control (Control).


$$Growth_{i,t}=a_{0}+a_{1}\mathrm{VC}+\sum_{k}\beta_{j}Control_{i,t}+{\mathrm{Year}}_{t}+{\mathrm{Ind}}_{i}+\varepsilon_{i,t}$$ (1)

$$Growth_{i,t}=a_{0}+a_{1}\mathrm{VC}*Cont+\sum_{k}\beta_{j}Control_{i,t}+Year_{t}+{\mathrm{Ind}}_{i}+\varepsilon_{i,t}$$ (2)

## Results

### Descriptive statistics and correlation analysis

Table 1 Shows the mean value and standard deviation of the main variables and their correlations. The correlation coefficients between variables are all less than 0.5, and there is no multicollinearity.

**Table 1:** Descriptive statistics and correlation analysis.

Variables	Mean	SD	Salgcha	Emplcha	Incomcha	VC	Seperati	Borderpr
Salgcha	0.721	0.365	1					
Emplcha	-0.162	2.891	0.425***	1				
Incomcha	0.181	0.436	0.065***	0.071***	1			
VC	0.379	0.485	0.051***	0.036***	-0.0100	1		
Seperati	0.118	0.176	-0.0100	-0.0200	0.00400	-0.102***	1	
Borderpr	-0.079	0.220	-0.0170	-0.026*	0.0120	0.096***	-0.343***	1
Size	21.256	0.904	0.00100	0.0190	0.00400	-0.087***	0.112***	-0.188***
Age	2.786	0.267	-0.0130	-0.0180	-0.030**	-0.158***	0.065***	-0.141***
Roe	0.260	0.102	0.151***	0.143***	0.040***	0.028***	0.028**	0.033**
Lev	0.319	8.502	0.032**	-0.0100	-0.031**	-0.100***	0.104***	-0.170***
Lnmansha	12.435	18.092	-0.037**	-0.00100	-0.00700	0.105***	-0.265***	0.711***
Numdir	5.190	1.253	0.00300	0.0110	0.00500	0.0180	0.068***	-0.142***
Variables	Mean	SD	Size	Age	Roe	Lev	Lnmansha	Numdir
Size	21.256	0.904	1					
Age	2.786	0.267	0.107***	1				
Roe	0.260	0.102	-0.133***	-0.087***	1			
Lev	0.319	8.502	0.365***	0.142***	-0.152***	1		
Lnmansha	12.435	18.092	0.0210	-0.079***	0.0130	-0.075***	1	
Numdir	5.190	1.253	0.089***	0.070***	0.031**	0.105***	-0.066***	1

Note

*p<0.10

**p< 0.05

***p<0.01.

### Analysis of hypothesis test results

#### The effect of venture capital participation on the performance of family enterprises

Through a multiple regression test, this paper discusses the influence of venture capital equity investment on the performance of the target enterprises.

First, a basic regression was conducted for family enterprises and non-family enterprises, respectively, to compare and analyse the impact of venture capital on the growth performance of family enterprises. The dependent variables are the growth rate of sales revenue, the growth rate in the number of employees, and the growth rate of net profit, and the independent variable is the risk capital. The regression results are shown in Table 2. In family enterprises (Panel A), venture capital is negatively correlated with their growth performance measurement index. Among them, the regression coefficients of the growth rate in the number of employees and venture capital in the direct regression and with the addition of control variables are -0.085 and -0.240, respectively, which are significantly negative at the levels of 1% and 10%, respectively. The regression coefficients of the growth rate of sales revenue and venture capital in the direct regression and with the addition of control variables are -0.012 and -0.011, respectively, and the growth rates of net profit are -0.001 and -0.002, respectively. Although the figures are not significant, the direction of the coefficient is consistent with the growth rate of sales revenue and the growth rate in the number of employees. To some extent, the regression results show that venture capital investment will hinder the growth and performance of family enterprises. However, in non-family enterprises, venture capital is positively correlated with growth performance. The growth rate of sales revenue is positively correlated with the regression coefficients of the direct regression and with the control variables of venture capital, which are 0.041 and 0.002, respectively, at the levels of 1% and 10%, respectively. The growth rate in the number of employees was positively correlated with the regression coefficients of the direct regression and with the addition of the control variables of venture capital, which were 0.028 and 0.013, respectively, at the levels of 1% and 10%, respectively. The correlation coefficients between the net profit growth rate and venture capital are 0.003 and 0.002, respectively, which are not significant but positive. The regression results show that venture capital intervention can promote the growth and performance of non-family enterprises. As a favourable social force, venture capital can encourage enterprises to carry out capital socialization transformation, which is very suitable for the development of enterprises in the growth stage. However, venture capital has an opposite effect on the growth of family enterprises, and its positive effect on family enterprises is weaker than its effect on non-family enterprises. One possible reason for this result is related to family enterprises’ preferences concerning venture capital equity financing because such an enterprise’s owners tend not to accept external equity investment. Therefore, after the introduction of venture capital, various conflicts will arise between the family enterprises and the venture capital management, resulting in the performance of the family enterprises being lower than that of the non-family enterprises. Thus, research hypothesis 2b is supported.

**Table 2:** Regression test of venture capital effect on family enterprise growth performance.

Panel A Family Sample Regression Results						
Variables	Salgcha	Salgcha	Emplcha	Emplcha	Incomcha	Incomcha
VC	-0.012	-0.011	-0.085***	-0.240*	-0.001	-0.002
	(1.06)	(-1.03)	(-3.12)	(-1.75)	(-1.30)	(-0.78)
Size		-0.066***		0.066***		-0.066***
		(-3.23)		(-3.23)		(-3.23)
Age		-0.138***		-0.135***		-0.137***
		(-2.59)		(-2.54)		(-2.57)
Roe		-0.851		-0.813***		-0.818***
		(-4.95)		(-4.70)		(-4.75)
Lev		0.423***		-0.422***		-0.422***
		(-4.35)		(-4.42)		(-4.42)
Lnmansha		0.196***		0.019***		0.019
		(2.77)		(2.65)		(1.38)
Numdir		0.016		0.016		0.016
		(1.36)		(1.36)		(1.38)
_cons	0.766***	2.442	0.776	2.448	0.763	2.450***
	(6.41)	(0.45)	(6.29)	(0.46)	(6.51)	(5.47)
Obs	843	843	843	843	843	843
Adj-R^2^	0.008	0.100	0.007	0.099	0.007	0.099
Panel B Non-Family Sample Regression Results						
Variables	Salgcha	Salgcha	Emplcha	Emplcha	Incomcha	Incomcha
VC	0.041***	0.002*	0.028***	0.013*	0.003	0.002
	(3.93)	(1.78)	(2.74)	(1.82)	(0.75)	(0.72)
Size		-0.066***		-0.024***		-0.023***
		(-3.23)		(-2.30)		(-2.26)
Age		-0.137***		-0.210***		-0.210***
		(-2.57)		(-7.08)		(7.10)
Roe		-0.818***		-0.538***		-0.017***
		(-4.75)		(-5.62)		(-5.41)
Lev		-0.422***		-0.210***		-0.209***
		(-4.42)		(-4.42)		(-4.38)
Lnmansha		0.019***		0.021***		0.021***
		(2.62)		(5.94)		(5.92)
Numdir		0.016		0.033***		0.033***
		(1.38)		(5.51)		(5.52)
_cons	0.435***	2.450***	0.439***	1.181***	0.444***	1.173***
	(6.30)	(5.47)	(6.48)	(5.05)	(6.86)	(5.02)
Obs	3152	3152	3152	3152	3152	3152
Adj-R2	0.002	0.099	0.001	0.045	0.045	0.045

Note: In parentheses is the value of T

*p<0.10

**p< 0.05

***p<0.01.

#### Moderating effect of family control degree

As shown in Table 3, the regression analysis demonstrates that the product of venture capital and the degree of family control over the venture capital and the two rights’ separation degree shown through the regression coefficient in a separate regression and joined along with the excess control returns the coefficients of 0.010 and 0.012, respectively, which are significant at the 10% level and negative. Using the two rights’ separation degree to measure the degree of family control on the performance of venture capital and the growth of family enterprises, there is a positive moderating role.

**Table 3:** State adjustment effect test of family control rights.

Variables	Growth	Growth	Growth	Growth
**VC**	-0.061	-0.095	-0.054	-0.144
	(-1.57)	(-0.84)	(0.39)	(0.90)
VC*Seperati		-0.010*		-0.012*
		(-1.79)		(-1.78)
VC*Borderpro			-0.003	-0.001
			(-0.11)	(-1.44)
**Size**	0.001	-0.007	-0.006	-0.013
	(0.01)	(-0.10)	(-0.08)	(0.19)
**Age**	0.146	0.157	0.136	0.156
	(0.86)	(0.92)	(0.80)	(0.91)
**Roe**	2.233***	2.267***	2.240***	2.279***
	(4.08)	(4.13)	(4.09)	(4.15)
**Lev**	0.671***	0.696***	0.689***	0.691***
	(2.21)	(2.28)	(2.25)	(2.22)
**Lnmansha**	-0.068	-0.074***	-0.070***	-0.071***
	(-3.03) ***	(-3.24)	(-2.85)	-(2.86)
**Numdir**	-0.001	-0.002	-0.001	-0.002
	(-0.03)	(-0.06)	(-0.02)	(-0.06)
**_cons**	1.178***	0.241	0.208	0.311
	(17.79)	(0.17)	(0.14)	(0.21)
**Obs**	840	840	840	840
**Adj-R2**	0.031	0.031	0.030	0.041

Note: In parentheses is the value of T

*p<0.10

**p< 0.05

***p<0.01.

In family enterprises, the greater the separation between control right and cash flow right, the more obvious the negative effect of venture capital on the growth performance of family enterprises. The higher the degree of family control, the more significant the inhibitory effect of venture capital on the growth performance of family enterprises. Although the degree of family control as measured by the index of excess control of board seats is not significant, the influence direction is the same as that of venture capital. Thus, research hypotheses 3 and 4 are partially supported.

### Robustness test

#### Endogeneity problem

This paper has empirically tested the inhibitory effect of venture capital intervention on the growth performance of family enterprises. However, venture capital impacts the growth performance of family enterprises in the first place because the influence of screening or post-investment activities inhibits the growth performance of family enterprises. This endogeneity problem may result in questioning of the empirical results, so this paper adopts the propensity score matching (PSM) method to solve the endogeneity problem.

In order to control the difference in venture capital shareholding samples, we extracted the matching group from the risk-free capital-backed family enterprises to form a new sample and then carried out regression on the model again. The specific approach is as follows.

The first step was to predict the behaviour of family enterprises when selecting venture capital and obtain the score of businesses’ propensities to choose venture capital. For each business, the risk-free business with the closest score in the same year and the same industry and the tendency score were selected as the paired sample, and the difference in the propensity score was within plus or minus 30%. Matching was carried out according to a 1:1 nearest matching method, and the tendency value in the experimental group was forcibly excluded to be greater than the maximum tendency value in the control group or lower than the minimum tendency value in the control group. The maximum distance allowed between the experimental group and the control group was 0.05. This also forced simultaneous recording when the observation group had more than one optimal match. The propensity score was estimated using the following logit model:

Logit(VC)=F(Size、Age、Roe、Lev、Lnmansha、Numdir)

The dependent variable is the possibility of acquiring venture capital. According to previous literature [39], the influencing factors, that is, the independent variables, include variables such as enterprise financial status, governance status, age, size, period, and industry, specifically size, age, Roe, Lev, Lnmansha, and Numdir. The PSM score analysis established a regression equation with the group variable (VC) as the dependent variable and each factor variable as the independent variable for logit regression. The propensity score of each company was calculated through the psmatch2 command, and a risk-free capital background company with the closest propensity score was matched for each company with a venture capital background. This score represents the probability that a venture capital firm will select the invested company, and these non-venture-capital-backed companies will constitute the control company group. The findings are shown in Table 4.

**Table 4:** Regression of risk capital selection influencing factors.

VC	Coef.	Std. Err.	z	P>|z|
Size	0.11	0.51	0.22	0.83
Age	-0.49	1.11	-0.45	0.66
Roe	-0.04	0.04	-1.06	0.29
Lev	0.04	0.02	1.92	0.06
Lnmansha	-0.45	0.22	-2.04	0.04
Numdir	-0.50	0.28	-1.81	0.07
_cons	13.61	9.44	1.44	0.15

The second step was to test whether the matched samples meet the common supporting hypothesis required by the PSM method. The results of variable checking to see whether the propensity score match is valid are presented in Table 5. The results show that the experimental group and the control group were similar under the same influencing factors, indicating that the PSM was effective.

**Table 5:** Matching balance test results.

Variables	Salgcha			Emplcha			Incomcha		
	EG	CG	Devi	EG	CG	Devi	EG	CG	Devi
Size	21.11	21.15	-2.9	21.16	20.72	3.4	21.16	21.22	3.4
Age	2.74	2.75	-3.8	2.74	2.77	-1.2	2.74	2.73	-1.2
Roe	10.77	9.93	6.5	10.91	7.11	3.1	10.91	10.11	3.1
Lev	24.55	23.98	3.6	25.19	25.26	3.3	25.19	25.26	3.3
Lnmansha	17.37	17.30	3.9	17.39	17.24	1.8	17.39	17.34	3.8
Numdir	5.57	5.83	-8.6	5.62	5.50	8.4	5.62	5.59	2.4

Note: EG, CG, and Devi mean the experimental group, the control group, and the deviation.

The third step was to use the matched samples to re-regress through the following model and compare and analyse whether the regression results are consistent with the main regression results.


$$Growth_{i,t}=a_{o}+a_{1}\mathrm{VC}+\sum_{k}\beta_{j}Control_{i,t}+{\mathrm{Year}}_{t}+{\mathrm{Ind}}_{i}+\varepsilon_{i,t}$$

First, calculate each sample to be chosen for venture capital and the enterprise probability value (propensity score). Then according to the nuclear nearest distance standard, the family enterprise with no risk capital background whose probability value is closest to each enterprise with venture capital participation is searched, and a total of 561 sample points are obtained. The above model was reused for regression, and the results are shown in Table 6. After matching, the influence of venture capital on the growth performance of the family enterprise is still significantly negative, which is consistent with the above regression results.

**Table 6:** Regression after score matching.

Variables	Salgcha	Emplcha	Incomcha
VC	-0.246*	-0.205*	-0.003
	(1.86)	(-1.95)	(1.07)
Size	-0.082***	-0.010**	-0.101
	(-2.85)	(-2.14)	(2.76) ***
Age	-0.526***	-0.136***	-0.101***
	(2.80)	(2.83)	(-2.91)
Roe	-0.010***	-0.708***	-0.808***
	(-2.78)	(-3.91)	(-2.99)
Lev	0.193**	-0.366**	-0.395**
	(3.08)	(3.18)	(2.79)
Lnmansha	0.103***	0.012***	0.012
	(2.75)	(2.08)	(1.61)
Numdir	0.026	-0.012	0.015
	(0.67)	(0.88)	(1.64)
_cons	2.762	2.771	2.762
	(0.29)	(0.39)	(3.61)
Obs	561	561	561
Adj-R2	0.065	0.089	0.087

Note: In parentheses is the value of T

*p<0.10

**p< 0.05

***p<0.01.

#### Robustness test of moderating effect

In order to further enhance the reliability and validity of the empirical analysis, the proxy variable of control rights was used to change the regulatory effect of control rights, and a robustness test of the regulatory effect was carried out based on the proportion of control right of the listed company owned by the actual controller (Cont). In addition, for the accuracy of the conclusion, the robustness test from this section is replaced with three proxy variables of growth performance, and the results are shown in Table 7. The proportion belonging to the actual controller who owns the control right of the listed company significantly increases the negative influence of venture capital on the growth rate of sales revenue. The empirical results of the sample data partially verify the robustness of the hypothesis.

**Table 7:** Test of moderating effect of degree of control right of replacement agent indicator.

Variables	Salgcha	Salgcha	Emplcha	Emplcha	Incomcha	Incomcha
VC	-0.099	-0.105	-0.024	-0.020	-0.002	-0.002
	(-1.03)	(-0.84)	(-0.75)	(-0.61)	(-0.78)	(-0.69)
VC* Cont		-0.010***		-0.001		-0.001
		(-3.17)		(-0.91)		(-0.95)
Size	-0.005	-0.008	-0.066***	-0.067***	-0.066***	-0.068***
	(-0.01)	(-0.13)	(-3.23)	(-3.24)	(-3.23)	(-3.23)
Age	0.097	0.088	-0.136***	-0.135***	-0.137***	-0.137***
	(0.65)	(0.58)	(-2.54)	(-2.50)	(-2.57) ***	(-2.53)
Roe	1.537***	1.443***	-0.813***	-0.794***	-0.818	-0.796***
	(3.16)	(2.96)	(-4.7)	(-4.55)	(-2.57)	(-4.59)
Lev	0.540***	0.667***	-0.422***	-0.431***	-0.422***	-0.431***
	(2.00)	(2.44)	(-4.42)	(-4.44)	(-4.42)	(-4.45)
Lnmansha	-0.067***	-0.075***	0.019***	0.019***	0.019***	0.019***
	(-3.38)	(-3.75)	(2.65)	(2.68)	(2.62)	(2.65)
Numdir	0.000	0.012	0.016	0.014	0.016	0.014
	(0.00)	(0.60)	(1.36)	(1.17)	(1.38)	(1.19)
_cons	0.571	0.784	2.449***	2.517***	2.450***	2.520***
	(0.45)	(0.60)	(5.46)	(5.42)	(5.47)	(5.43)
Obs	833	833	833	833	833	833
Adj-R^2^	0.023	0.031	0.099	0.099	0.099	0.099

Note: In parentheses is the value of T

*p<0.10

**p< 0.05

***p<0.01.

## Conclusions

### Theoretical contribution

First, this study enriches our knowledge of the role of venture capital in corporate growth. Previous research has revealed the effect of venture capital on corporate growth [40–42]. However, there is a lack of relevant research on the organizational form of family enterprises. Previous research has revealed the non-financial resources, such as empirical knowledge and international resources, that venture capital brings to family businesses [8]. Venture capital has a positive effect on family business performance [5]. Venture capital firms that hold a minority stake in a family enterprise negatively affect performance [43]. We found that research concerning venture capital financing on family enterprise growth performance has not reached a consensus. The decision-making control mechanism is important to the enterprise and represents a type of internal environment that could promote or hinder the management process. This means that the unique decision-making control mechanism of family enterprises causes venture capital investment in these enterprises to yield different investment effects. Our study considered the characteristics of family control, which helps us to better understand the economic consequences of family enterprise venture capital financing and the family enterprise growth path supported by venture capital, thereby supplementing and enriching relevant research on the impact of venture capital on enterprise growth.

Second, this study provides insights into family enterprise venture capital financing based on the perspective of control power, which enriches the research on control power theory in the relationship between equity financing and corporate growth. Venture capital also has a relationship with the influence mechanism of and control power over growth performance. Existing research has mostly regarded family enterprises as a homogeneous economic entity, while ignoring the characteristics of heterogeneity between these enterprises. The related manifestation of this heterogeneity is the degree of family control. There are differences in the degree of enterprise control across different family enterprises, and the effects of family control vary as well [44]. Few previous studies have examined the effect mechanism of venture capital on family enterprises from the perspective of family control characteristics. By examining the role of the degree of family control in the venture capital financing growth performance, this study expands the research on the theory of control in the equity financing of family enterprises.

### Practical contribution

First, current research provides supporting data and a decision-making basis for family businesses to better use venture capital financing. The introduction of venture capital by family enterprises may break the original family-led control environment of the decision-making entities and form a decision-making mechanism for diversified decision-making entities. The unique control mechanism of family enterprises makes the heterogeneity of the influence of venture capital closely related to this decision-making mechanism. From the perspective of control rights, this research expounds on how they affect family enterprise venture capital financing and explores the interpretation path of the negative impact of venture capital on family enterprise growth performance. The research reveals the mechanism of venture capital’s impact on the effectiveness of family enterprise growth performance and provides supporting evidence and practical reference for family enterprise venture capital financing.

Second, the study can inspire venture capital investors (investment institutions) to rationally anticipate and deal with their relationships with family enterprises when they invest in the actual management of the enterprises. Venture capital participation in family enterprises has a negative effect on their growth performance and is influenced by the heterogeneity of the degree of control of family enterprises. This finding is enlightening to the relationship between rational expectation and coping with family enterprises when venture capital firms invest in the actual management of the enterprises. For example, according to the degree of family control, we should rationally deal with the problems that may arise between the family enterprises and avoid losses as much as possible.

### Research limitations and suggestions

First, the control power factors that affect family enterprise venture capital financing are relatively complicated, and the theoretical models and empirical evidence of the influence of family control power remain in the exploratory stage. This study simplifies the power types of control rights. The theory and model need to be further refined and more robustly verified. However, the degree of control was affected by the life cycle status of family enterprises: depending, for example, on whether family enterprises were at the founding family stage or the descendant family stage and on whether the founding family was the first-generation principal or the second-generation co-manager. Future research could further expand knowledge of the manifestation of the degree of control and more accurately provide empirical support for the effective use of venture capital by family enterprises, especially for family enterprises that are inheriting.

Second, it was difficult to obtain relevant data for this study. Although it takes a lot of time to manually collect information on variables such as family enterprises and the degree of family control and then try to confirm everything found, there may be inaccuracies caused by incomplete information and the difficult collection methods. The degree of the data’s usefulness is limited. In addition, there is a need for further systematization to measure growth performance. Future research can look for authoritative databases through which to obtain the necessary data.

## Supporting information

S1 File (XLSX)
